# Micromechanisms and Characterization of Low-Velocity Impact Damage in 3D Woven Composites

**DOI:** 10.3390/ma15196636

**Published:** 2022-09-24

**Authors:** Jin Sun, Yunfeng Dai, Linhai Huang, Diantang Zhang, Junhua Zhao

**Affiliations:** 1 Jiangsu Key Laboratory of Advanced Food Manufacturing Equipment and Technology, Jiangnan University, Wuxi 214122, China; 2 Key Laboratory of Eco-Textiles, Ministry of Education, Jiangnan University, Wuxi 214122, China

**Keywords:** carbon fibers, woven composites, impact behavior, finite element analysis

## Abstract

Low-velocity impact (LVI) damage of 3D woven composites were experimentally and numerically investigated, considering different off-axis angles and impact energies. The impact responses were examined by LVI tests, and the damage morphology inside the composites was observed by X-ray micro-computed tomography (*μ*-CT). Yarn-level damage evolution was revealed by developing a hybrid finite element analysis model. The results show that the impact damage has significant directionality determined by the weft/warp orientation of the composites. The damage originates at the bottom of the impacted area and then expands outwards and upwards simultaneously, accompanied by in-plane and out-of-plane stress transfers. The straight-line distributed weft/warp yarns play an important role in bearing loads at the beginning of loading, while the w-shape distributed binder warp yarns gradually absorb impact deformation and toughen the whole structure as the loading proceeds. The effect of directional impact damage on post-impact performance was explored by performing compressing-after-impact (CAI) tests. It is revealed that the CAI properties along principal directions are more sensitive to the low-velocity impact, and the damage mode is significantly affected by the loading direction.

## 1. Introduction

Composite materials have been increasingly applied in the aerospace, wind energy, and automobile industries as functional construction materials due to their high strength, low density, and flexible designability [1,2,3,4,5,6]. However, the composite structures in service are susceptible to low-velocity impact damage resulting from hail stone, dropping tools, runway sand, etc. The damage is difficult to detect since it often leaves no visible indication on the surface. This undetectable damage can continuously grow in the subsequent use, leading to a decline in mechanical properties and potential failure [7,8,9,10,11,12]. Three-dimensional woven composites can effectively overcome the delamination behavior depending on the through-thickness binder yarns in comparison with traditional laminated composites, and their impact resistance and damage tolerance are improved [13,14,15,16,17,18,19,20]. Comparative studies on the impact resistance of 2D and 3D woven composites show that 3D woven composites can absorb more impact energy, producing a smaller pit depth and damage area, and their delamination length and opening are less than those of 2D woven composites due to the suppression of z-yarns [21].

The failure mechanisms of 3D woven composites under low-velocity impact have recently attracted widespread attention. A lot of experiments have been carried out to investigate the impact behavior from the aspects of impact response and damage morphology by employing nondestructive examination technologies such as ultrasonic C-scan, X-ray micro-computed tomography (*μ*-CT), digital image correlation (DIC), acoustic emission (AE), infrared thermography (IRT), etc. [22,23]. Seltzer et al. [24] conducted low-velocity impact tests on 3D woven composites reinforced with various component materials. With the help of X-ray microtomography, it was found that the impact strength of the materials was primarily dependent on the in-plane fiber breakage, while their energy absorption capability was mainly affected by the presence of z-yarns, which could delay delamination and maintain the structural integrity, promoting energy dissipation by tow splitting, intensive fiber fracture under the impactor, and the formation of a plug by out-of-plane shear. By comparison, 3D woven composites dissipated over twice the energy of 2D laminates. Ralph et al. [25] revealed that minor changes in binder float-length can significantly influence both out-of-plane and in-plane impact performance of 3D woven composites. Out-of-plane drop-weight impact showed the increases in float-length declined energy absorption in warp direction with no significant changes in weft, while higher float length showed higher crush force efficiency and specific energy absorption in axial impact tests. In both out-of-plane and axial impact scenarios, higher float length can increase damage tolerance. Wang et al. [26] explored the dynamic damage resistance of 3D woven carbon/heterocyclic aramid fiber hybrid composites subjected to low-velocity impact. The results showed that the fiber hybrid form had a great influence on the impact response and that the intralaminar hybrid 3D woven composite showed the best comprehensive performance and was found to have stable initial stiffness as well as impact resistance. Some scholars evaluated the impact resistance of 3D woven composites by paying attention to their post-impact mechanical response. Hart et al. [27] investigated both compression-after-impact (CAI) and flexure-after-impact (FAI) properties of 2D and 3D woven glass/epoxy composites. Post-impact flexural strength and modulus from FAI tests showed larger reductions with respect to impact energy in comparison to CAI results. Three-dimensional woven composites architecturally retained greater post-impact mechanical performance due to the through-thickness z-tow, which suppressed delamination growth and opening during impact.

Numerical simulation is becoming more and more effective for investigating the impact behavior of 3D woven composites [28,29]. During the numerical modeling, the homogenization method based on unit-cell and the full-size meso-structural model are often employed, and the constitutive model, failure criteria, and degradation model after damage initiation for component materials should be determined. Shah et al. [30] proposed a multiscale progressive damage modeling methodology for 3D woven composites, which can be implemented in most finite element software to create a digital twin for simulation of damage response. Zhang et al. [31] numerically investigated the impact damage behaviors of a 3D angle-interlock woven composite subjected to transverse impact at subzero temperatures. A coupled thermo-mechanical constitutive model has been developed to capture the effects of an increase in temperature, strain rate sensitivity, and fragmentation of the composite material. With the help of numerical simulation, Wu et al. [32] analyzed the synergistic effect of 3D orthogonal woven structure and asymmetric carbon/glass hybridization on impact response. Yarn-level finite element models, including failure criteria and progressive damage law, were developed to compare the structural deformation and damage behavior. In recent years, the multi-scale hybrid analysis model has been developed to simulate various mechanical behaviors of 2D and 3D textile composites [33,34]. The meso-structure and homogenized models are respectively assigned for the region of concern and other areas, comprehensively considering the precision and efficiency of the calculation. Cao et al. [35] established a multi-scale finite element model based on the ductile and shear damage criteria, combining a micro-structure model with a continuum model, to simulate 3D angle-interlock woven composites subjected to low-velocity impact, and the LVI damage distributions were unveiled.

Three-dimensional woven composites are anisotropic materials with orthogonal yarn configurations comprising straight-line and w-shape distributed yarns, hence their failure mechanisms under low-velocity impact are complicated. It is possible that different types of yarns can play different roles in bearing loads, and the damage degree of material may vary in different directions, which can further influence the post-impact performance. Many previous studies on low-velocity impact behaviors of composites focused on the effects of structural parameters, impact condition, and environmental factors, such as material thickness, impactor shape, and temperature, but few of them considered and evaluated the directionality of the impact damage and its influence on the post-impact performance. In the existing numerical simulations, the yarn-level damage evolutions of different component yarns, which are actually helpful in revealing the damage mechanism of composites, were rarely reported. In this paper, the failure mechanism of 3D woven composites subjected to low-velocity impact was studied by experimental and numerical methods. The standard test method using rectangular specimens of a particular size was employed to conduct low-velocity impact (LVI) and compression after impact (CAI) tests, and the specimens with various given off-axis angles were prepared to realize the compression along different directions. The variation of the off-axis angle changed the length and distribution of yarns inside the specimen, causing the different off-axis angle specimens to have a certain degree of boundary effect during the impact. From the LVI tests, the impact responses under various off-axis angles and impact energies were obtained. The assessment of response results proved that the boundary effect could be neglected, and the post-impact properties of different off-axis angle specimens were comparable. Based on this, the interior damage morphology and failure mode were observed using the *μ*-CT scanning, and a hybrid finite element analysis model was established to reveal the yarn-level damage evolution, especially for various component yarns. The effect of directional impact damage on post-impact performance was examined by the CAI tests, and the specimen damage in corresponding failure modes was discussed with the help of the full-field strain distribution on the specimen surface measured by the DIC technology.

## 2. Experiment

### 2.1. Materials

Three-dimensional angle-interlock woven composites are made of high-performance carbon fibers T700-12K supplied by Toray Inc. (Chuo-ku, Japan), reinforcing E-51 epoxy resin. The mechanical properties of component materials are shown in Table 1. The woven preforms contain 4 layers of weft yarns, 3 layers of binder warp yarns, and 3 layers of warp yarns, and all yarns have a density of 1.8 g/cm^3^. The orthogonally oriented weft and warp yarns are distributed in straight lines, and the binder warp yarns interlock with the weft layers in a wavy form along the warp direction. The binder warp and warp yarns are alternately arranged with a ratio of 2:1. The epoxy resin was injected into the preforms by the RTM process to achieve the composite panels. The specific parameters of woven composites are listed in Table 2. Following the predesigned directions, the composite panels were finally cut into the specimens with a dimension of 150 mm × 100 mm.

### 2.2. Low-Velocity Impact Tests

The low-velocity impact tests were performed using an Instron Dynatup 9350HV drop-weight testing machine according to the standard test method ASTM D7136/D7136M-15. The hemispherical shape impactor was adopted with a diameter of 16 mm, and the total mass of the drop hammer combined with counterweight was 5.449 kg. The four corners of the specimen were attached to the hollowed fixture by rubber clamping tweezers, forming a free impact area of 125 mm × 75 mm, and the impact point was located in the specimen center. Five off-axis angles (0°, 30°, 45°, 60°, and 90°) and two impact energies (21.6 J and 47.6 J) were considered, and three specimens were prepared for each working condition.

### 2.3. Compressive Tests after Impact

CAI tests were conducted using a universal testing machine, following the ASTM D7137/D7137M-17 standard. The loading rate was set to 1 mm/min, and the load-displacement curves and maximum loads were recorded.

## 3. Numerical Simulation

### 3.1. Low-Velocity Impact Model

**Figure 1:**
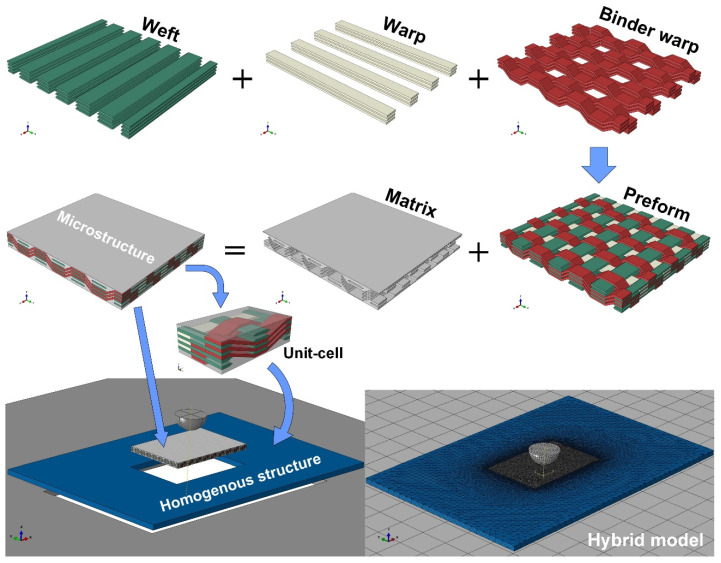
Hybrid finite element analysis model.

Three-dimensional woven composites possess periodic architectures. The representative volume element (RVE), namely the unit-cell, is usually established to study this type of material. The unit-cell model can effectively predict the stiffness of the material, saving plenty of computational resources, but the predicted strength properties are difficult to reach with high accuracy, and the damage evolution of the whole material cannot be predicted. The full-size meso-structural model can resolve the above problem but has high requirements for the computational conditions. In this work, a hybrid analysis model was established to simulate the low-velocity impact behaviors of 3D woven composites, as shown in Figure 1. Considering the relatively thin thickness of the material, a full-thickness unit-cell is established instead of the divided surface and interior unit-cells. The area around the impact point is built by the meso-structure obtained by arraying the unit-cells, and the surrounding area far away from the impact loading is considered as the homogenization model whose mechanical properties are characterized by those of a single unit-cell. The meso-structure area can describe yarn-level mechanical response and damage in detail, while the homogenized area where there is nearly no damage and little deformation plays an important role in transferring load and displacement. The hybrid model cannot only reflect the details of damage but also decrease the cost of calculation. The size of the whole model is 150 mm × 100 mm, and the meso-structure part is 48 mm × 30 mm. The meso-structure area and the homogenized area are connected by a tie constraint, so that the stress and deformation can be smoothly transferred. The impactor and fixture base are regarded as rigid bodies in the simulation.

Considering the structural complexity of the hybrid model, the 4-node linear tetrahedron element (C3D4) is assigned to the specimen with fine meshes (seed density is 0.25) for the meso-structure area and coarse meshes (seed density is 4) for the homogenized area. The element number of different off-axis angle models is slightly biased. Taking the 0° model as an example, the whole model consists of 971,670 elements, where 757,340 elements for the meso-structure model and 214,330 for the continuum model. The 8-node reduced integral solid element (C3D8R) is adopted for mesh generation of the impactor and fixture base, and their mesh numbers are 1280 and 380, respectively.

### 3.2. Material Model

Both the meso-structure and the single unit-cell model characterizing the homogenized structure contain two component materials: resin matrix and fiber bundles. Their material behaviors, including constitutive relations, damage initiation, and damage evolution, are described below.

#### 3.2.1. Resin Matrix

The matrix is considered to be isotropic, conforming to the von Mises yield criterion [35]. Once the stress of a matrix element meets the criterion, its stiffness will be directly degraded by multiplying by the reduction coefficient.

#### 3.2.2. Fiber Bundles

The fiber bundles impregnated with epoxy resin are regarded as a transversely isotropic material. The constitutive relation is expressed by:

(1) $$\sigma_{ij}=C_{ijkl}\varepsilon_{kl}$$

where $\sigma_{ij}$, $C_{ijkl}$, and $\varepsilon_{kl}$(i,j,k,l=1,2,3) denote engineering stress, stiffness matrix, and elastic strain components. The damage stiffness matrix is given as follows:

(2) $$C_{d}=\frac{1}{\Delta }\left[\begin{array}{cccccc} d_{f}E_{11}(1-d_{m}\nu_{23}\nu_{32}) & d_{f}d_{m}E_{11}(\nu_{21}+\nu_{23}\nu_{31}) & d_{f}E_{11}(\nu_{31}+d_{m}\nu_{21}\nu_{32}) & & & \\ & d_{m}E_{22}(1-d_{f}\nu_{13}\nu_{31}) & d_{m}E_{22}(\nu_{32}+d_{f}\nu_{12}\nu_{31}) & & & \\ & & E_{33}(1-d_{f}d_{m}\nu_{12}\nu_{21}) & & & \\ & & & \Delta d_{f}d_{m}S_{12} & & \\ & & & & \Delta d_{f}d_{m}S_{23} & \\ & & & & & \Delta d_{f}d_{m}S_{13} \end{array}\right]$$

in which $d_{f}$ and $d_{m}$ are the damage variables of fiber and matrix, calculated by:

(3) $$\{\begin{array}{c} d_{f}=(1-d_{ft})(1-d_{fc})\\ d_{m}=(1-S_{mt}d_{mt})(1-S_{mc}d_{mc})\\ \Delta =1-d_{f}d_{m}\nu_{12}\nu_{21}-d_{m}\nu_{23}\nu_{32}-d_{f}\nu_{13}\nu_{31}-2d_{f}d_{m}\nu_{21}\nu_{32}\nu_{13} \end{array}$$

where *d* is the damage variable, and the subscripts *ft*, *fc*, *mt,* and *mc* are the fiber tension, fiber compression, matrix tension, and matrix compression, respectively. The coefficients $S_{mt}=0.9$ and $S_{mc}=0.5$ are set to control the shear stiffness loss caused by the tensile and compressive damage of the matrix [36].

The strain-formed Hashin criterion is employed to predict the damage initiation of fiber bundles as follows:

Fiber tensile failure ($\varepsilon_{11}\geq 0$)

(4) $$F_{ft}={\left(\frac{\varepsilon_{11}}{\varepsilon_{ft}^{0}}\right)}^{2}\geq 1,\;\varepsilon_{ft}^{0}=\frac{X^{T}}{E_{11}}$$

Fiber compressive failure ($\varepsilon_{11}<0$)

(5) $$F_{fc}={\left(\frac{\varepsilon_{11}}{\varepsilon_{fc}^{0}}\right)}^{2}\geq 1,\;\varepsilon_{fc}^{0}=\frac{X^{c}}{E_{11}}$$

Matrix tensile failure ($\varepsilon_{22}+\varepsilon_{33}\geq 0$)

(6) $$F_{mt}={\left(\frac{\varepsilon_{22}}{\varepsilon_{mt}^{0}}\right)}^{2}\geq 1,\;\varepsilon_{mt}^{0}=\frac{Y^{T}}{E_{22}}$$

Matrix compressive failure ($\varepsilon_{22}+\varepsilon_{33}<0$)

(7) $$F_{mc}={\left(\frac{E_{22}\varepsilon_{22}+E_{33}\varepsilon_{33}}{2S_{12}}\right)}^{2}+\frac{\varepsilon_{22}+\varepsilon_{33}}{\varepsilon_{mc}^{0}}\left[{\left(\frac{E_{22}\varepsilon_{mc}^{0}}{2S_{12}}\right)}^{2}-1\right]+{\left(\frac{G_{23}}{S_{23}}\right)}^{2}(\varepsilon_{23}^{2}-\frac{E_{22}E_{33}}{G_{23}}\varepsilon_{22}\varepsilon_{33})+{\left(\frac{G_{12}\varepsilon_{12}}{S_{12}}\right)}^{2}+{\left(\frac{G_{13}\varepsilon_{13}}{S_{13}}\right)}^{2}\geq 1\varepsilon_{mc}^{0}=\frac{Y^{C}}{E_{22}}$$

where $\varepsilon_{ij}$ and $E_{ij}(i,j=1,2,3)$ are the elastic strain tensor and Young’s modulus, $\varepsilon_{I}^{0}(I=ft,fc,mt,mc)$ the initial failure strain, $X^{T}$ and $X^{C}$ the tensile and compressive strength in the axial direction of fiber bundles, $Y^{T}$ and $Y^{C}$ the tensile and compressive strength in the transverse direction of fiber bundles, and $S_{ij}$ the shear strength.

For further performance degradation, the damage evolution model based on fracture energy is adopted. The damage variables are given by:

(8) $$d_{I}=\frac{\varepsilon_{I}^{f}}{\varepsilon_{I}^{f}-\varepsilon_{I}^{0}}(1-\frac{\varepsilon_{I}^{0}}{\varepsilon_{I}})\;\left(d_{I}\in (0,1),I=ft,fc,mt,mc\right)$$

in which $\varepsilon_{I}^{f}$ is the final failure strain. When the corresponding damage variable reaches one, the strain can be calculated by Equations (9)–(12), respectively:

(9) $$\varepsilon_{ft}^{f}=\frac{2G_{ft}}{X^{T}l}$$

(10) $$\varepsilon_{fc}^{f}=\frac{2G_{fc}}{X^{C}l}$$

(11) $$\varepsilon_{mt}^{f}=\frac{2G_{mt}}{Y^{T}l}$$

(12) $$\varepsilon_{mc}^{f}=\frac{2G_{mc}}{Y^{C}l}$$

where $G_{I}(I=ft,fc,mt,mc)$ is the fracture energy density and l the characteristic length of the element.

## 4. Results and Discussion

### 4.1. Validation of Numerical Model

**Figure 2:**
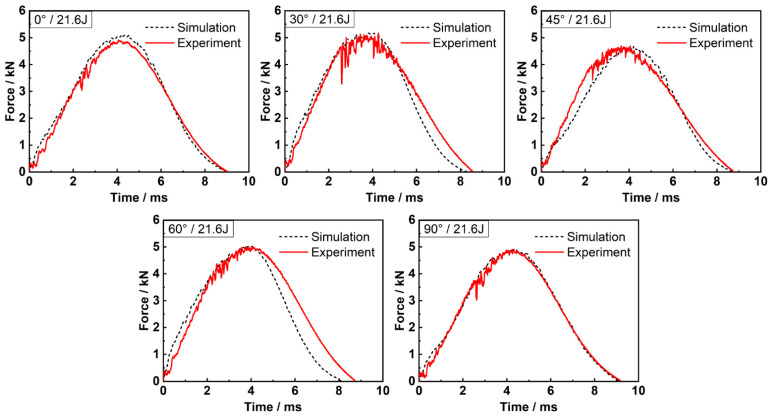
Force-time relations under 21.6 J of impact energy.

**Figure 3:**
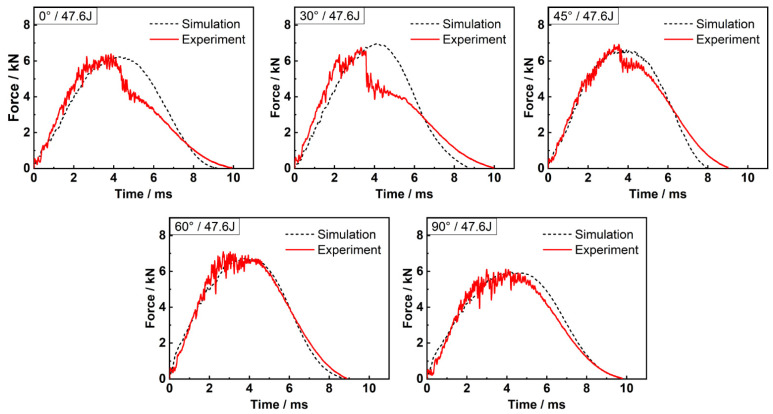
Force-time relations under 47.6 J of impact energy.

To validate the effectiveness and accuracy of the hybrid finite element analysis model, the simulated force-time responses under various off-axis angles and impact energies were compared with the experimental results, and a good agreement was achieved, as shown in Figure 2 and Figure 3. Table 3 shows the peak force, maximum displacement of impactor and contact duration obtained by experiment and simulation, respectively. The maximum error of −14.66% proves the numerical model reliable for this study.

### 4.2. Low-Velocity Impact Response

**Figure 4:**
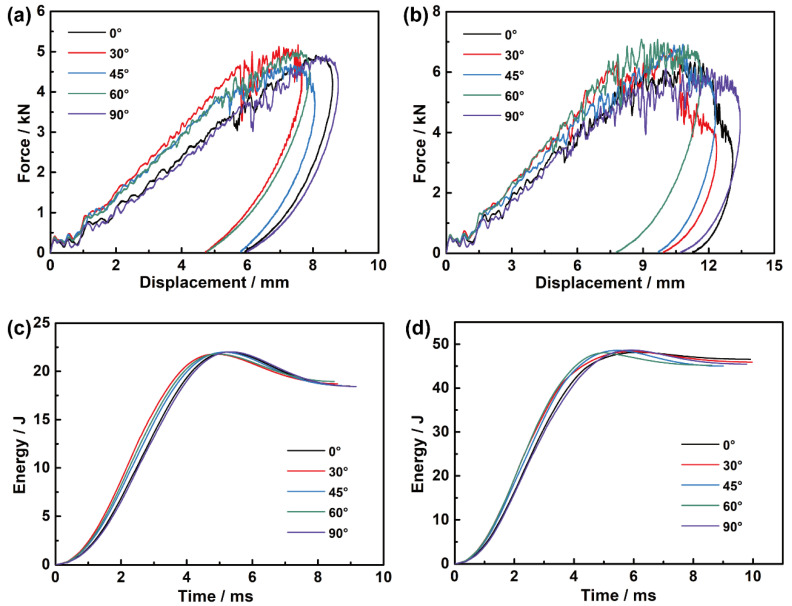
Impact responses: (**a**) force-displacement curves under 21.6 J of impact energy; (**b**) force-displacement curves under 47.6 J of impact energy; (**c**) energy-time curves under 21.6 J of impact energy; (**d**) energy-time curves under 47.6 J of impact energy.

Figure 4a,b depict the force-displacement relations of the five off-axis angle specimens under the two impact energies. The maximum displacement is recorded when the velocity of the impactor is reduced to zero, and the permanent displacement is measured at the moment when the impactor leaves the surface of the specimen. Under each impact energy, the maximum/permanent displacements of the principal direction specimens are relatively larger than those of the off-axis specimens. The curves under the same energy have a close trend, and the higher energy brings a higher peak force and larger maximum/permanent displacement. All the curves show an initial continuous growth, followed by an approximate plateau, where different degrees of vibration are caused by the damage accumulation and continuous redistribution of loads, until the final rebound. The length and volume proportion of the fibers passing through the impact area of different angle specimens are distinct due to their rectangular shape, which leads to a certain discrepancy in mechanical response. Within the size range of the specimen, the longer fiber can contribute to higher flexural rigidity and peak force to the specimen. Consequently, the curves of off-axis specimens rise faster than those of principal direction specimens at first and reach a higher peak force. It is seen that the specimens with higher flexural rigidity and peak force produce relatively smaller maximum/permanent displacement, and then the area enclosed by each curve under the same impact energy is almost the same, which indicates that the adsorbed energies of various off-axis angle specimens under each energy are nearly identical.

Figure 4c,d show the energy-time curves under the two impact energies. Part of the kinetic energy of the impactor is transformed into the elastic energy of the specimen through contact deformation, while the other is absorbed by the damage, friction, and vibration of the panel. The mean values of the final absorbed energy and the energy absorption rate under 21.6 J of impact energy are 18.5 J and 85.6%, and those under 47.6 J are 45.5 J and 97.2%. It is seen that higher impact energy causes more energy absorption, and the absorbed energies of various angle specimens under the same impact energy are very close.

Overall, the discrepancy in impact responses of different off-axis angle specimens is quite limited, and the impact-conducted damage, friction, and deformation of various angle specimens under the same impact energy reflected in the final energy absorption are extremely similar. Based on this, it can be considered that the boundary effect caused by the shape and size of specimens can be neglected, and their post-impact properties are comparable.

### 4.3. Impact Damage Analysis

**Figure 5:**
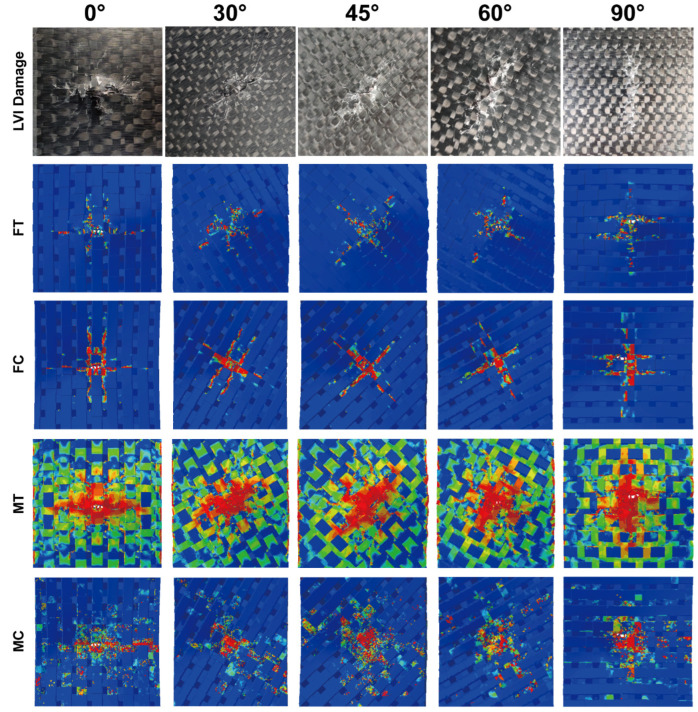
Surface damage morphology under 47.6 J of impact energy obtained by LVI tests and FEM: fiber tensile damage (FT), fiber compressive damage (FC), matrix tensile damage (MT), and matrix compressive damage (MC).

**Figure 6:**
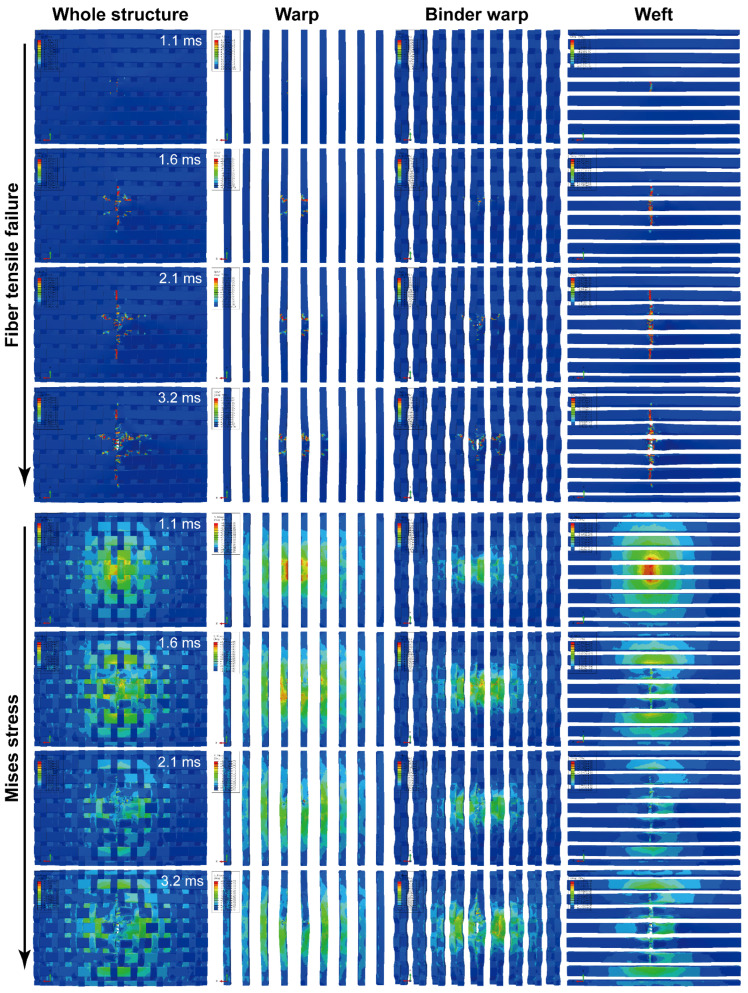
Damage evolutions and corresponding stress distributions of the whole structure and component yarns for a 0° specimen under 47.6 J of impact energy.

**Figure 7:**
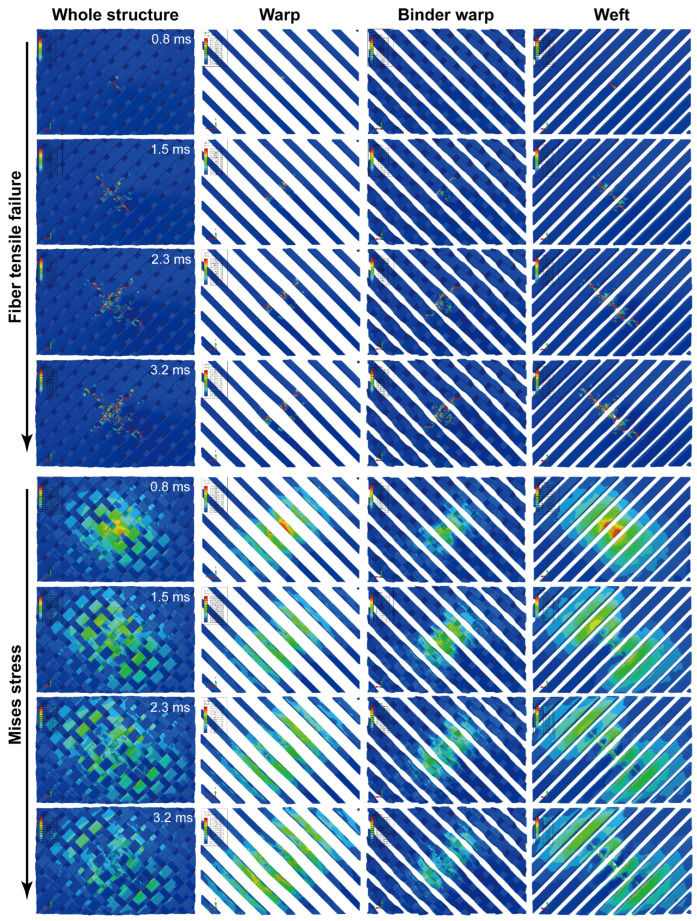
Damage evolutions and corresponding stress distributions of the whole structure and component yarns for a 45° specimen under 47.6 J of impact energy.

**Figure 8:**
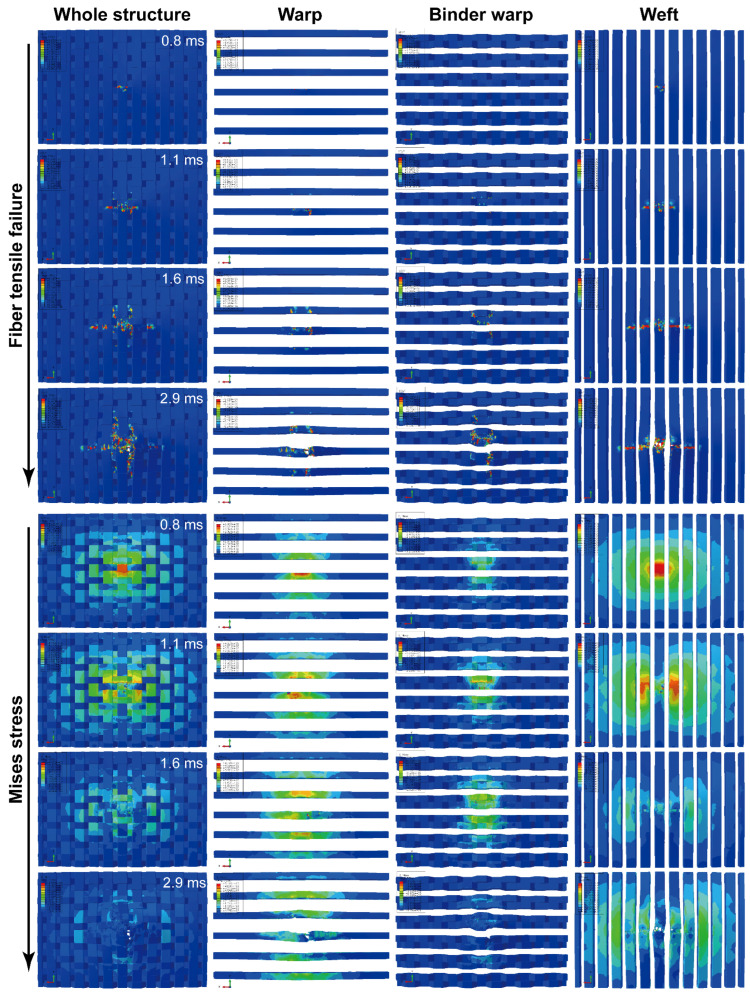
Damage evolutions and corresponding stress distributions of the whole structure and component yarns for a 90° specimen under 47.6 J of impact energy.

Considering the more severe damage can be demonstrated relatively more clearly, the impact damage under 47.6 J of impact energy will be analyzed. Figure 5 depicts surface damage morphologies under 47.6 J of impact energy, and the tensile/compressive failures of fiber and matrix on the back/front side of the specimen are presented. The distribution of these damages changes regularly with the off-axis angle, and their extension directions are always consistent with the warp/weft orientation. Taking the 0°, 45°, and 90° specimens as an example, Figure 6, Figure 7 and Figure 8 show the damage evolutions and corresponding stress distributions of the whole structure and component yarns under 47.6 J of impact energy. At the beginning of the loading, the straight-line distributed weft and warp yarns bear the main impact loads, and always first produce stress concentrations and initial damage in the impacted area. As the loading proceeds, the intensifying damage reduces the carrying capacity of these yarns, and their stress is gradually transferred to the yarns on both sides, where the new damage can be produced. This leads to a phenomenon where the damage of weft/warp yarns expands along the warp/weft direction, forming a cruciform distribution. However, the binder warp yarns with a w-shaped distribution can absorb a certain bending deformation, and their load capacity is gradually mobilized. Hence, their tensile damage appears later, and the stress level shows a certain upward trend. In addition, their tensile damage appears mainly on the back side (stretched side) of the specimen, which can give an explanation why more warp-direction distributed damage can be found on the back of the specimen. It can be concluded from the above that the impact damage of the material has obvious directionality, which may further affect its residual properties along different directions.

**Figure 9:**
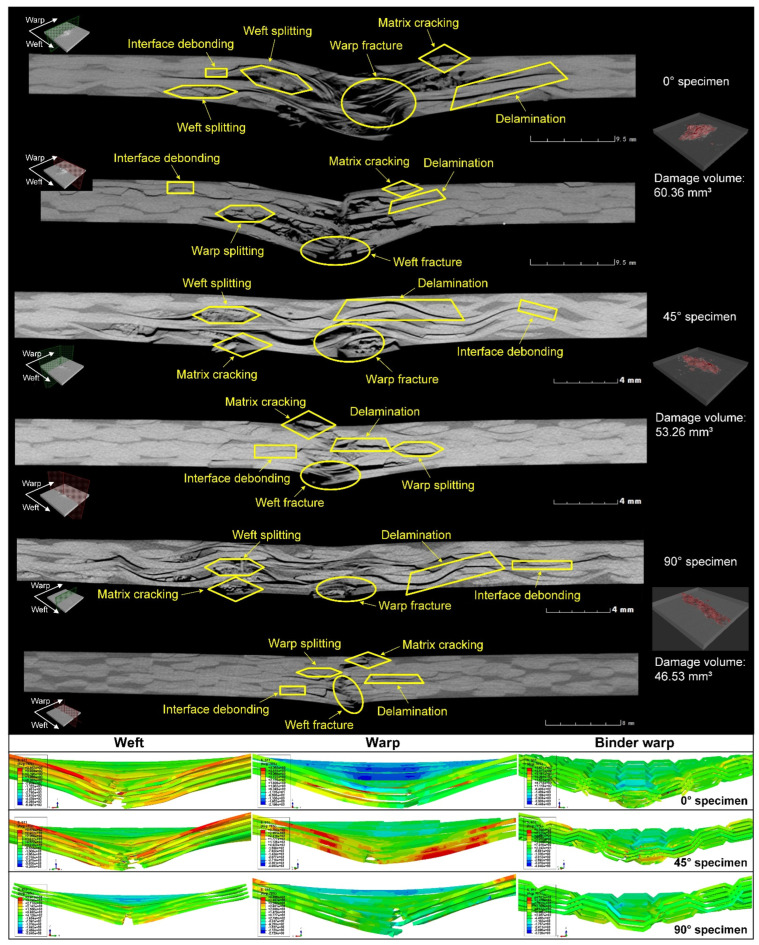
Internal damage morphology and axial stress distribution of the component yarns under 47.6 J of impact energy.

To observe the internal damage of impacted specimens, X-ray micro-computed tomography (*μ*-CT) was employed. The cross-section slices of the specimen are respectively parallel to the warp and weft directions. Figure 9 shows the scanning results of 0°, 45°, and 90° specimens under 47.6 J of impact energy, combined with the axial stress distribution of component yarns at the maximum displacement of the impactor. Impact damage goes through the whole thickness of the specimen, and the damage area is larger on the back side. A number of fiber bundles in the impacted area are completely fractured, and in the surrounding area there is significant delamination accompanied by a few crushed fibers caused by the large deformation and mutual squeeze of yarns. Meanwhile, the damage from interface debonding, matrix cracking, and fiber splitting can also be observed. No detectable cracks appear immediately underneath the impactor contact zone, which indicates that the cracks are the result of shear or tensile stresses, rather than compressive ones [24]. With the help of image processing, the damage volume of the speimen was reconstructed and calculated quantitatively. The damage volume declines slightly with the increase of the off-axis angle since the growing angle improves the length and volume proportion of the binder warp yarns passing through the impact region, which plays a role in absorbing bending deformation and resisting impacts. It is seen from the stress nephogram that due to the bending deformation caused by the impact, the specimens are subjected to different degrees of tension on the back side, where the yarn fracture always first appears, while certain compressive stresses are produced in the impacted region of the front side. The bearing capacity of the fractured yarns decreases, and with the redistribution of the loads, the tensile stress is transferred upwards to the adjacent yarns where the new damage or fracture may be produced. As a result, the yarn fractures are produced from the bottom up. Combined with Figure 6, Figure 7 and Figure 8, it can be seen that as the impact damage develops, the in-plane and out-of-plane stress transfers occur simultaneously. The stress level of binder warp yarns under each impact energy decreases as the off-axis angle increases. This is because the growing angle raises the length and volume proportion of binder warp yarns passing through the impact area, which improves the capability to absorb deformation, disperse loads, and decrease the stress concentration. The lower stress just leads to slighter damage, thus the larger the off-axis angle, the less the damage, which is consistent with the *μ*-CT scanning results. It can be concluded that the binder warp yarns can toughen the whole structure to resist the impact.

### 4.4. CAI Properties

**Figure 10:**
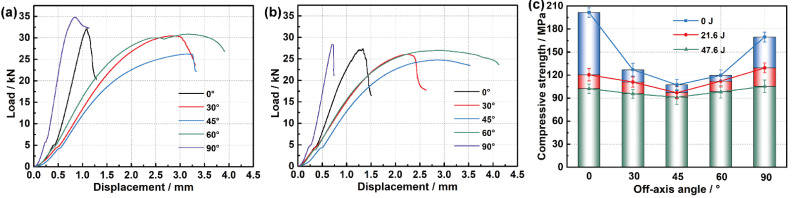
CAI responses: (**a**) load-displacement curves under 21.6 J of impact energy; (**b**) load-displacement curves under 47.6 J of impact energy; (**c**) CAI strength under various impact energies.

Figure 10a,b demonstrate the load-displacement curves of the five off-axis angle specimens under the two impact energies, respectively. The curves of principal direction specimens increase almost linearly before reaching their peak value, and then decline sharply until their failure, exhibiting a certain brittle failure characteristic. By contrast, the curves of off-axis direction specimens increase linearly at first, followed by nonlinear growth to the peak value, and then drop gradually, showing some ductile failure. Under 21.6 J of impact energy, the principal direction curves have obviously higher initial slopes and peak values with smaller failure displacements, while the 45° curve has the lowest initial slope and peak value. A similar situation also appears under 47.6 J of impact energy, but the maximum values of various curves decline at different degrees and show smaller deviations from each other. In addition, the peak values of off-axis direction curves occur relatively earlier.

Figure 10c shows the CAI strength along various directions. The studied 3D woven composites are anisotropic, hence their compressive responses along different off-axis directions are distinct, even though they are not impacted. In view of the fact that the change of compressive strength along various off-axis directions caused by the impact rather than the configuration of the material itself is the main concern, not only the value of compressive strength under each impact energy but also its variation amplitudes against the strength of the unimpacted specimens need to be discussed. As a contrast, the static strength of the unimpacted specimens is also measured and displayed as CAI strength under 0 J impact energy in the figure. The CAI strength under each impact energy always declines first and then grows with the increase of the off-axis angle, leaving a minimum value at a 45° angle. Compared with the compression strengths of the unimpacted specimens, the values of 0°, 30°, 45°, 60°, and 90° specimens under 21.6 J of impact energy are respectively 40.3%, 12.6%, 9.6%, 6.2%, and 23.7% lower, and the values under 47.6 J of impact energy decrease by 49.3%, 24.7%, 14.9%, 18.0%, and 37.9%. As the impact energy increases, the compressive strength along principal directions drops more noticeably than that along off-axis directions, and the closer to the 45° direction, the smaller the decline is, which indicates that the CAI properties along principal directions are more sensitive to the low-velocity impact.

**Figure 11:**
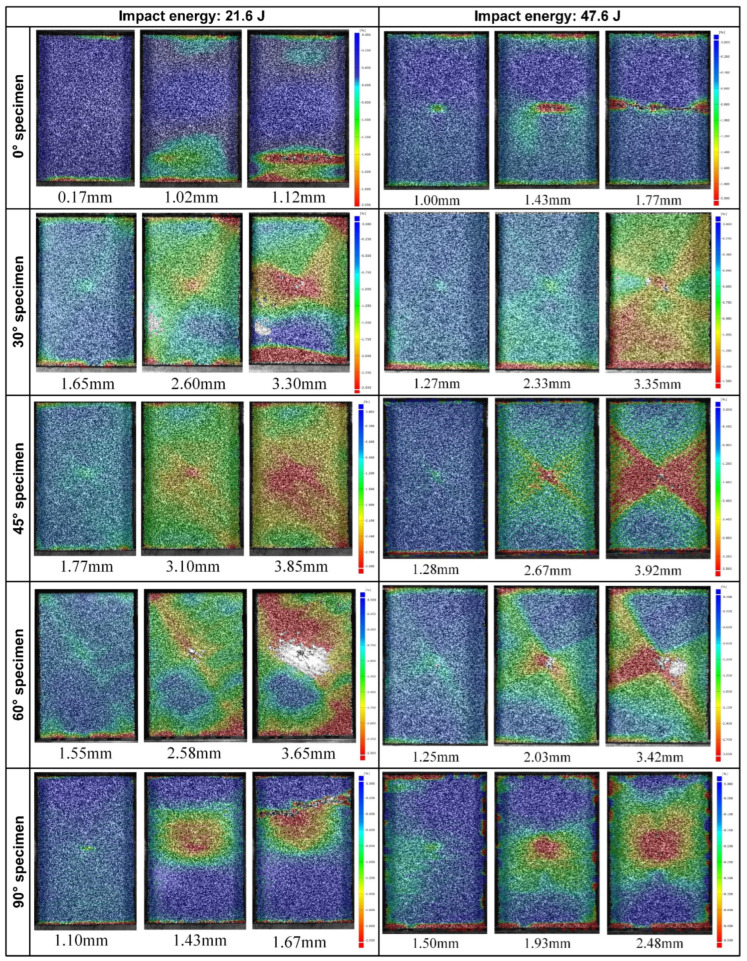
Full-field strain distribution on the front of the specimens under corresponding compression displacement.

Digital Image Correlation (DIC) was employed to measure the full-field strain distribution on the specimen surface. The strain distribution on the front of specimens at corresponding compression displacement is depicted in Figure 11. Inside 0° and 90° specimens, there exists a certain amount of fibers distributed along the loading direction, which are the main bearing structure, and when their compressive stress reaches a certain level, a sudden crushing failure is produced, leaving a relatively small failure displacement and a line-distributed damage area parallel to the specimen edge. However, the stress concentration and damage of these two off-axis angle specimens under 21.6 J of impact energy are not located at the impacted area like those under 47.6 J of impact energy but almost cover the whole width near the end of the specimens. This is because, at such a low impact energy, the locations of CAI damage are more affected by the clamping of the fixture than by the relatively slight impact damage. The high strain of the other off-axis angle specimens appears at the original impact area and extends along the weft/warp direction as the off-axis angle changes. For these specimens, the carrying capacity of fibers is not fully mobilized due to the deviation between fiber orientation and loading direction. However, the off-axis loading brings the specimens more obvious shear action and puts them under a pressure-shear coupled stress state, producing significant stress concentration and damage along the weft/warp direction. As the compression loads increase, the damage is continuously accumulated until the final failure of specimens, leaving a relatively large failure displacement, and it is difficult for the fibers to reach a high axial stress level to cause specimens a sudden crushing failure like 0° and 90° specimens.

## 5. Conclusions

The damage mechanism of 3D woven composites under low-velocity impact was experimentally and numerically studied. The main conclusions are summarized as follows:

1. The experimental observation and numerical simulation show that the impact damage always extends along weft yarns, warp yarns, or binder warp yarns, indicating that the damage has obvious directionality determined by weft/warp orientation;
2. The impact damage begins at the bottom of the impacted area and then expands outwards and upwards simultaneously, accompanied by in-plane and out-of-plane stress transfers. The carrying order and damage degree of various component yarns are different. The straight-line distributed weft/warp yarns bear most of the loads at the beginning of loading, whose stress concentrations and damage are produced relatively early, while the w-shape distributed binder warp yarns can absorb a certain impact deformation, whose carrying capacity is gradually mobilized. The binder warp yarns play an important role in toughening the whole structure to resist impact;
3. The effect of impact damage on residual compression performance was analyzed by performing CAI tests. The closer to the principal directions, the more sensitive the CAI strength is to the impact. The damage mode is significantly influenced by the loading direction. The principal direction specimens show certain brittle failure characteristics, while the off-axis direction specimens exhibit some ductile failure.

## Figures and Tables

**Table 1:** Mechanical properties of component materials.

Property	T700 Carbon Fiber	E-51 Epoxy Matrix
Elastic modulus (GPa)	Longitudinal: *E*_f1_ = 232	*E*_m_ = 3.5
	Transverse: *E*_f2_ = 15	
Shear modulus (GPa)	Longitudinal: *G*_f12_ = 24	*G*_m_ = 1.296
	Transverse: *G*_f23_ = 5.03	
Poisson’s ratio	*μ*_f12_ = 0.28	*μ*_m_ = 0.35
Tension strength (MPa)	*X*_ft_ = 4850	*S*_mt_ = 80
Compression strength (MPa)	*X*_fc_ = 2470	*S*_mc_ = 241
Shear strength (MPa)	\	*S*_ms_ = 60

**Table 2:** Detailed parameters of the 3D angle-interlock woven composites.

Sample Name	Fabric Parameters				Composites Parameters		Impact Energies (J)
	Number of Layers	Warp Density (tows/cm)	Binder Warp Density (tows/cm)	Weft Density (tows/cm)	Thickness (mm)	Fiber Volume Fractions (%)	
0° sample	4	5	5	2	2.67	45.8	21.6
	4	5	5	2	2.67	45.8	47.6
30° sample	4	5	5	2	2.75	47.2	21.6
	4	5	5	2	2.75	47.2	47.6
45° sample	4	5	5	2	2.71	46.5	21.6
	4	5	5	2	2.71	46.5	47.6
60° sample	4	5	5	2	2.75	47.2	21.6
	4	5	5	2	2.75	47.2	47.6
90° sample	4	5	5	2	2.69	46.1	21.6
	4	5	5	2	2.69	46.1	47.6

**Table 3:** Comparison of numerical and experimental results.

Sample	*F*_max_ (kN)		Error (%)	*Dis*_max_ (mm)		Error (%)	*t* (ms)		Error (%)
	Exp.	FEM		Exp.	FEM		Exp.	FEM	
0°/21.6 J	4.91	5.10	3.87	8.60	8.11	−5.70	8.97	9.00	0.33
30°/21.6 J	5.17	5.18	0.19	7.66	6.71	−12.40	8.56	8.33	−2.69
45°/21.6 J	4.69	4.67	−0.43	8.06	7.94	−1.49	8.76	8.67	−1.03
60°/21.6 J	5.03	4.98	−0.99	7.89	7.07	−10.39	8.73	8.14	−6.76
90°/21.6 J	4.91	4.89	−0.41	8.77	8.10	−7.64	9.18	9.17	−0.11
0°/47.6 J	6.40	6.23	−2.66	13.10	11.37	−13.21	9.92	9.03	−8.97
30°/47.6 J	6.75	6.92	2.52	12.36	11.10	−10.19	9.97	8.78	−11.94
45°/47.6 J	6.93	6.67	−3.75	12.31	10.96	−10.97	9.01	7.98	−11.43
60°/47.6 J	7.09	6.72	−5.22	11.62	10.71	−7.83	8.87	8.68	−2.14
90°/47.6 J	6.12	5.98	−2.29	13.44	11.47	−14.66	9.76	9.87	1.13

*F*_max_ is peak force, *Dis*_max_ is maximum displacement of the impactor, and *t* is total contact duration.

## References

[1] Ge X.X., Zhang P., Zhao F., Liu M., Liu J., Cheng Y.S. (2022). Experimental and numerical investigations on the dynamic response of woven carbon fiber reinforced thick composite laminates under low-velocity impact. Compos. Struct..

[2] Banik A., Zhang C., Khan M.H., Wilson M., Tan K.T. (2022). Low-velocity ice impact response and damage phenomena on steel and CFRP sandwich composite. Int. J. Impact Eng..

[3] Wang A., Wang X.J., Xian G.J. (2021). The influence of stacking sequence on the low-velocity impact response and damping behavior of carbon and flax fabric reinforced hybrid composites. Polym. Test..

[4] Choi J.I., Park S.E., Nguyen H.H., Lee Y., Lee B.Y. (2022). Resistance of hybrid layered composite panels composed of fiber-reinforced cementitious composites against high-velocity projectile impact. Compos. Struct..

[5] Long S.C., Chen C., Wang H.R., Yao X.H., Zhang X.Q. (2022). Distribution and propagation of matrix cracks within composite laminates under impact. Compos. Struct..

[6] Kuteneva S.V., Gladkovsky S.V., Vichuzhanin D.I., Nedzvetsky P.D. (2022). Microstructure and properties of layered metal/rubber composites subjected to cyclic and impact loading. Compos. Struct..

[7] Cao W.J., Wu Y.Y., Sun B.Z., Gu B.H., Hu M.Q. (2022). Impact crack quantification analyses in 3-D angle-interlock woven composite using image segmentation method. Eng. Fract. Mech..

[8] Falcó O., Lopes C., Sommer D., Thomson D., Ávila R., Tijs B. (2022). Experimental analysis and simulation of low-velocity impact damage of composite laminates. Compos. Struct..

[9] Kazemianfar B., Nami M.R. (2021). Influence of oblique low velocity impact on damage behavior of 2D and 3D woven composites: Experimental and numerical methods. Thin-Walled Struct..

[10] Yang W.C., Huang R.X., Liu J.Y., Liu J.X., Huang W. (2022). Ballistic impact responses and failure mechanism of composite double-arrow auxetic structure. Thin-Walled Struct..

[11] Deng Y.F., Zeng X.Z., Wang Y.T., Du J., Zhang Y.B. (2022). Research on the low-velocity impact performance of composite sandwich structure with curved-crease origami foldcore. Thin-Walled Struct..

[12] Ekici R., Kosedag E., Demir M. (2022). Repeated low-velocity impact responses of SiC particle reinforced Al metal-matrix composites. Ceram. Int..

[13] Munoz R., Seltzer R., Sket F., Gonzalez C., Llorca J. (2022). Influence of hybridization on energy absorption of 3D woven composites under low-velocity impact loading. Model. Exp. Valid. Int. J. Impact Eng..

[14] Li Z.J., Sun B.Z., Gu B.H. (2010). FEM simulation of 3D angle-interlock woven composite under ballistic impact from unit cell approach. Comput. Mater. Sci..

[15] Shah S.Z.H., Yusoff P.S.M.M., Karuppanan S., Choudhry R.S., Din I.U., Othman A.R., Sharp K., Gerard P. (2021). Compression and buckling after impact response of resin-infused thermoplastic and thermoset 3D woven composites. Compos. Part B.

[16] Elias A., Laurin F., Kaminski M., Gornet L. (2017). Experimental and numerical investigations of low energy/velocity impact damage generated in 3D woven composite with polymer matrix. Compos. Struct..

[17] Han D., Jia X., Zhang H.J., Gao X.G., Han X., Sun L., Zheng Z.K., Zhang L., Wang F., Song Y.D. (2022). Foreign object damage and post-impact tensile behavior of plain-woven SIC/SIC composites. Compos. Struct..

[18] Wang X., Hu B., Feng Y., Liang F., Mo J., Xiong J., Qiu Y. (2008). Low velocity impact properties of 3D woven basalt/aramid hybrid composites. Compos. Sci. Technol..

[19] Ahmed S., Zheng X.T., Yan L.L., Zhang C., Wang X. (2020). Influence of asymmetric hybridization on impact response of 3D orthogonal woven composites. Compos. Sci. Technol..

[20] Kazemianfar B., Esmaeeli M., Nami M.R. (2020). Response of 3D woven composites under low velocity impact with different impactor geometries. Aerosp. Sci. Technol..

[21] Hart K.R., Chia P.X.L., Sheridan L.E., Wetzel E.D., Sottos N.R., White S.R. (2017). Mechanisms and characterization of impact damage in 2D and 3D woven fiber-reinforced composites. Compos. Part A.

[22] Tuo H.L., Lu Z.X., Ma X.P., Xing J., Zhang C. (2019). Damage and failure mechanism of thin composite laminates under low-velocity impact and compression-after-impact loading conditions. Compos. Part B.

[23] Zhang D.T., Sun M.Y., Liu X.D., Xiao X.L., Qian K. (2019). Off-axis bending behaviors and failure characterization of 3D woven composites. Compos. Struct..

[24] Seltzer R., Gonzalez C., Munoz R., Lorca J.L., Varela T.B. (2013). X-ray microtomography analysis of the damage micromechanisms in 3D woven composites under low-velocity impact. Compos. Part A.

[25] Ralph C., Dahale M., Neale G., Ramaswamy K., McCarthy M., Yoo S., Toso N., Kelly J., McGarrigle C., Archer E. (2021). Influence of binder float length on the out-of-plane and axial impact performance of 3D woven composites. Compos. Part A.

[26] Wang C.Z., Su D.D., Xie Z.F., Zhang K., Wu N., Han M.Y., Zhou M. (2021). Low-velocity impact response of 3D woven hybrid epoxy composites with carbon and heterocyclic aramid fibres. Polym. Test..

[27] Hart K.R., Chia P.X.L., Sheridan L.E., Wetzel E.D., Sottos N.R., White S.R. (2017). Comparison of Compression-After-Impact and Flexure-After-Impact protocols for 2D and 3D woven fiber-reinforced composites. Compos. Part A.

[28] Zhao Q.L., Wang W.H., Liu Y.T., Hou Y.L., Li J.S., Li C. (2022). Multiscale modeling framework to predict the low-velocity impact and compression after impact behaviors of plain woven CFRP composites. Compos. Struct..

[29] Zhang X.F., Xiao Y.R., Meyer C.S., O’Brien D.J., Ghosh S. (2022). Impact damage modeling in woven composites with two-level Parametrically-Upscaled Continuum Damage Mechanics Models (PUCDM). Compos. Part B.

[30] Shah S.Z.H., Yusoff P.S.M.M., Karuppanan S., Choudhry R.S., Sajid Z. (2021). Multiscale damage modelling of 3D woven composites under static and impact loads. Compos. Part A.

[31] Zhang J.J., Zhang W., Huang S.W., Gu B.H. (2021). An experimental-numerical study on 3D angle-interlock woven composite under transverse impact at subzero temperatures. Compos. Struct..

[32] Wu Z.Y., Zhang L.C., Ying Z.P., Ke J., Hu X.D. (2020). Low-velocity impact performance of hybrid 3D carbon/glass woven orthogonal composite: Experiment and simulation. Compos. Part B.

[33] Patel D.K., Waas A.M., Yen C.F. (2018). Direct numerical simulation of 3D woven textile composites subjected to tensile loading: An experimentally validated multiscale approach. Compos. Part B.

[34] Patel D.K., Waas A.M., Yen C.F. (2019). Compressive response of hybrid 3D woven textile composites (H3DWTCs): An experimentally validated computational model. J. Mech. Phys. Solids.

[35] Cao W.J., Zhang J.J., Sun B.Z., Gu B.H. (2019). X-ray tomography and numerical study on low-velocity impact damages of three-dimensional angle-interlock woven composites. Compos. Struct..

[36] Liao B.B., Liu P.F. (2017). Finite element analysis of dynamic progressive failure of plastic composite laminates under low velocity impact. Compos. Struct..

